# Comparison of volumetric dynamic optical coherence tomography with biological methods for evaluation of radiation effects in prostate tumor spheroids

**DOI:** 10.1117/1.JBO.31.10.106004

**Published:** 2026-10-13

**Authors:** Steph Swanson, Keyu Chen, Elahe Cheraghi, Ernest Osei, Kostadinka Bizheva

**Affiliations:** a University of Waterloo, Science, Department of Physics and Astronomy, Waterloo, Ontario, Canada; b Waterloo Regional Health Network, Department of Medical Physics, Kitchener, Ontario, Canada; c University of Guelph, Science, Department of Clinical Studies, Guelph, Ontario, Canada; d University of Waterloo, Engineering, Systems Design Engineering Department, Waterloo, Ontario, Canada; e University of Waterloo, Science, School of Optometry and Vision Sciences, Waterloo, Ontario, Canada

**Keywords:** optical coherence tomography, dynamic contrast optical coherence tomography, radiation, clonogenic assay, tumor spheroid, agent-based modeling

## Abstract

**Significance:**

Three-dimensional (3D) tumor spheroids are more physiologically representative of *in vivo* patient tumors compared with two-dimensional monolayer culture. However, their 3D nature challenges the use of conventional biological techniques such as proliferation assays, fluorescence microscopy, and the clonogenic assay, which is the gold standard method for assessing cell survival following radiation. However, the clonogenic assay requires spheroid disaggregation.

**Aim:**

Non-invasive volumetric imaging with dynamic optical coherence tomography (dOCT) enables cellular activity to be visualized with spatial resolution within 3D tumor spheroids. Cellular activity observed via dOCT in irradiated prostate tumor spheroids was quantified for comparison with conventional biological techniques.

**Approach:**

A Varian TrueBeam linear accelerator was used to irradiate spheroid and monolayer cultures with a 6-MV beam. Cellular activity was estimated from dOCT images generated via frequency banding and compared with clonogenic assay, proliferation assay, fluorescence microscopy, and 3D cell simulation.

**Results:**

Prostate cancer cells cultured as spheroids demonstrated improved radio-resistance via clonogenic assay compared with monolayer culture. The dOCT method demonstrated quantitative and qualitative agreement with the proliferation assay and fluorescence microscopy, respectively.

**Conclusions:**

A longer duration of repeated dOCT measurement in tumor spheroids following radiation treatment could offer a non-invasive alternative to the clonogenic assay.

## 1 Introduction

Prostate cancer is the most prevalent cancer in North American males^1^^,^^2^ and is commonly treated with radiation therapy.^3^ Clinically, modern radiation treatments are delivered through fractionation schemes that leverage distinct radiobiological behavior exhibited by different tumor types and their surrounding tissues.^4^^–^^6^ Our foundational radiobiological understanding of cellular responses to radiation was formed through conventional two-dimensional (2D) monolayer cell culture methods.^7^^,^^8^ In 1956, Puck and Marcus^9^ irradiated mammalian cells cultured in 2D monolayers and published the first radiation survival curve that fit cell survival to radiation dose using a colony-forming or clonogenic assay. Since then, the clonogenic assay has remained the gold standard for measuring radiation survival,^10^ in part because it informs the extensively validated linear–quadratic (LQ) model.^11^^,^^12^ In the LQ model, the linear α and quadratic β parameters mechanistically describe cell killing due to one or two radiation tracks, respectively.^13^ The α/β ratios obtained from conventional 2D monolayer cell culture experiments continue to guide clinical decision-making.^7^ However, a growing body of evidence in recent decades has demonstrated that *in vitro* cells cultured in 2D monolayers on flat, hard surfaces do not accurately replicate the behavior of *in vivo* tumors.^14^^,^^15^ In contrast, cancer cells cultured in small three-dimensional (3D) aggregates called tumor spheroids exhibit more physiologically relevant behavior due to increased cell-to-cell contact and diffusion-limited distributions of oxygen and nutrients.^16^^–^^18^ This heterogeneous distribution leads to the formation of a hypoxic spheroid core of non-proliferative or quiescent cells.^15^ It has been widely observed that radiation survival differs significantly between *in vitro* cancer cells cultured in 2D monolayers and those cultured in 3D spheroids.^19^^–^^21^

Cell culture geometry is suspected to influence the effects of radiation through biological and physical processes related to nutrient and waste transport, resource competition, and mechanical forces exchanged between cancer cells and their microenvironment.^19^^,^^22^^,^^23^ Mathematical *in silico* models that simulate these biological and physical processes have become increasingly prevalent as tools to elucidate the mechanisms underlying tumor growth and treatment response.^24^^,^^25^ In particular, *in silico* methods have been utilized to investigate the mechanical and molecular characteristics of tumor spheroids.^26^ Conventional *in silico* approaches, such as continuum models described by partial differential equations (PDEs), are mathematically well-characterized and computationally efficient; however, these models struggle to accurately capture the inherent cellular heterogeneity and diversity present within patient tumors and tumor spheroids.^27^ In contrast, discrete *in silico* methods such as agent-based models address these limitations by simulating each cancer cell as an individual “agent” that evolves through time by responding to its local environment, including neighboring cells and nutrient availability.^24^^,^^28^ The utility of *in silico* models for falsifying hypotheses and generating novel insights depends critically upon the accuracy of their parameter estimates, which are derived from *in vitro* and/or *in vivo* measurements.^29^

However, the 3D structure of tumor spheroids poses challenges for measurement by conventional biological techniques. The clonogenic assay can be adapted for evaluating radiation survival in spheroid cultures by disaggregating the 3D cellular structures into a cell suspension for 2D plating and longitudinal observation of colony formation. Nevertheless, the geometry of cell culture following radiation treatment itself influences cellular survival and response.^20^ Alternative methods, such as proliferation assays, avoid the need for spheroid disaggregation and have also been utilized to estimate clonogenic radiation survival.^30^ For instance, the Alamar Blue proliferation assay is a common technique that measures cellular metabolism by detecting the reduction of non-fluorescent, blue resazurin to fluorescent pink resorufin.^31^ Although the AB proliferation assay has been adapted for spheroid cultures,^32^ the fluorescence measurement reflects only an averaged response across the entire cell population within the spheroid and does not distinguish the treatment effect in the hypoxic core from the spheroid periphery. Fluorescence microscopy (FM) is widely employed to evaluate spheroids with spatial resolution. Recent live-cell workflows combining fluorescence imaging, image analysis, and flow cytometry have enabled quantitative characterization of normoxic, hypoxic, and necrotic regions and their differential responses to irradiation.^33^ Bright-field image analysis has also been used to derive patient-derived organoid survival curves after X-ray irradiation.^34^ Among label-free metabolic approaches, optical redox imaging measures endogenous NAD(P)H and FAD autofluorescence and can quantify treatment-response heterogeneity at the individual-organoid level.^35^ Quantitative oblique back-illumination microscopy has likewise enabled longitudinal, label-free, high-resolution monitoring of therapy-induced cellular changes in glioblastoma spheroids, including responses to radiation.^36^ These approaches provide complementary readouts but differ in labeling requirements, biological specificity, spatial resolution, penetration depth, acquisition speed, and three-dimensional coverage.

Optical coherence tomography (OCT)^37^ provides label-free, depth-resolved volumetric imaging of intact tumor spheroids,^38^ whereas dynamic OCT (dOCT) additionally derives activity-related contrast from temporal OCT intensity fluctuations.^39^ Recently, dOCT has been employed to investigate spheroids^40^^–^^43^ as well as other types of 3D cell cultures^43^^–^^46^. Quantitative analysis of tumor spheroids using dOCT has been conducted following chemotherapy treatment^40^^,^^41^ and exposure to perfluorooctanoic acid.^43^ Although the clonogenic assay remains the gold standard for measuring cell survival after radiation exposure, there has been sustained interest in adapting alternative methods, such as proliferation assays, to circumvent the long experiment duration and labor-intensive colony counting required by clonogenic assays.^30^^,^^47^^,^^48^ Given prior suggestions that dOCT can quantify cellular activity and viability,^40^^,^^42^ it may also provide another feasible alternative to the clonogenic assay. In this study, we employed a previously described high-speed spectral-domain line-field (LF) dOCT system^49^ to image prostate tumor spheroids after radiation treatment and performed qualitative and quantitative comparisons with established biological methods and computational simulation.

## 2 Methods

### 2.1 Prostate Tumor Spheroids

PC3 cells (human prostate adenocarcinoma) were cultured in RPMI 1640 (Sigma-Aldrich, Saint Louis, Missouri, United States) supplemented with 1% penicillin (Thermo Fisher Scientific, Waltham, Massachusetts, United States) and 10% fetal bovine serum (Thermo Fisher Scientific). Cells were incubated at 37°C with 5% ${\mathrm{CO}}_{2}$ in a humidified atmosphere. Monolayer cell culture was plated in T-25 culture flasks. Prostate tumor spheroids were seeded and cultured in a 3D Petri Dish (MicroTissues Inc., Providence, Rhode Island, United States) composed of 2% agarose gel. Each agarose gel contained a 9×9 array of wells and was seeded to form spheroids composed of ∼1000 cells in each well [Fig. 1(a)].

### 2.2 Radiation Treatment

A Varian TrueBeam linear accelerator was used to deliver 1, 2, 4, 6, 8, or 10 Gy to monolayer and spheroid cultures with a 6-MV beam 24 h after seeding [Fig. 1(b)]. Each 6-well plate containing gels with spheroids was positioned at a source-to-axis distance (SAD) of 100 cm, at a depth of 5 cm within Gammex 457-CTG solid water, and surrounded by Superflab bolus for scatter conditions. Nonetheless, we expect a delivered dose with an uncertainty in the range of 10%. T-25 culture flasks of monolayer culture were similarly situated and treated. Live control spheroids, which were not exposed to radiation, were removed from incubation and handled identically to treated samples. At the time of treatment, a subset of live control spheroids were fixed with 4% formaldehyde (Thermo Fisher Scientific) in phosphate buffered saline (PBS) (Wisent Inc., Canada) for 1 h to eliminate metabolic activity. These fixed spheroids were subsequently stored at 4°C for ∼24  h before measurement to minimize dOCT motion artifacts due to the cross-linking induced by fixation.^50^

**Fig. 1:**
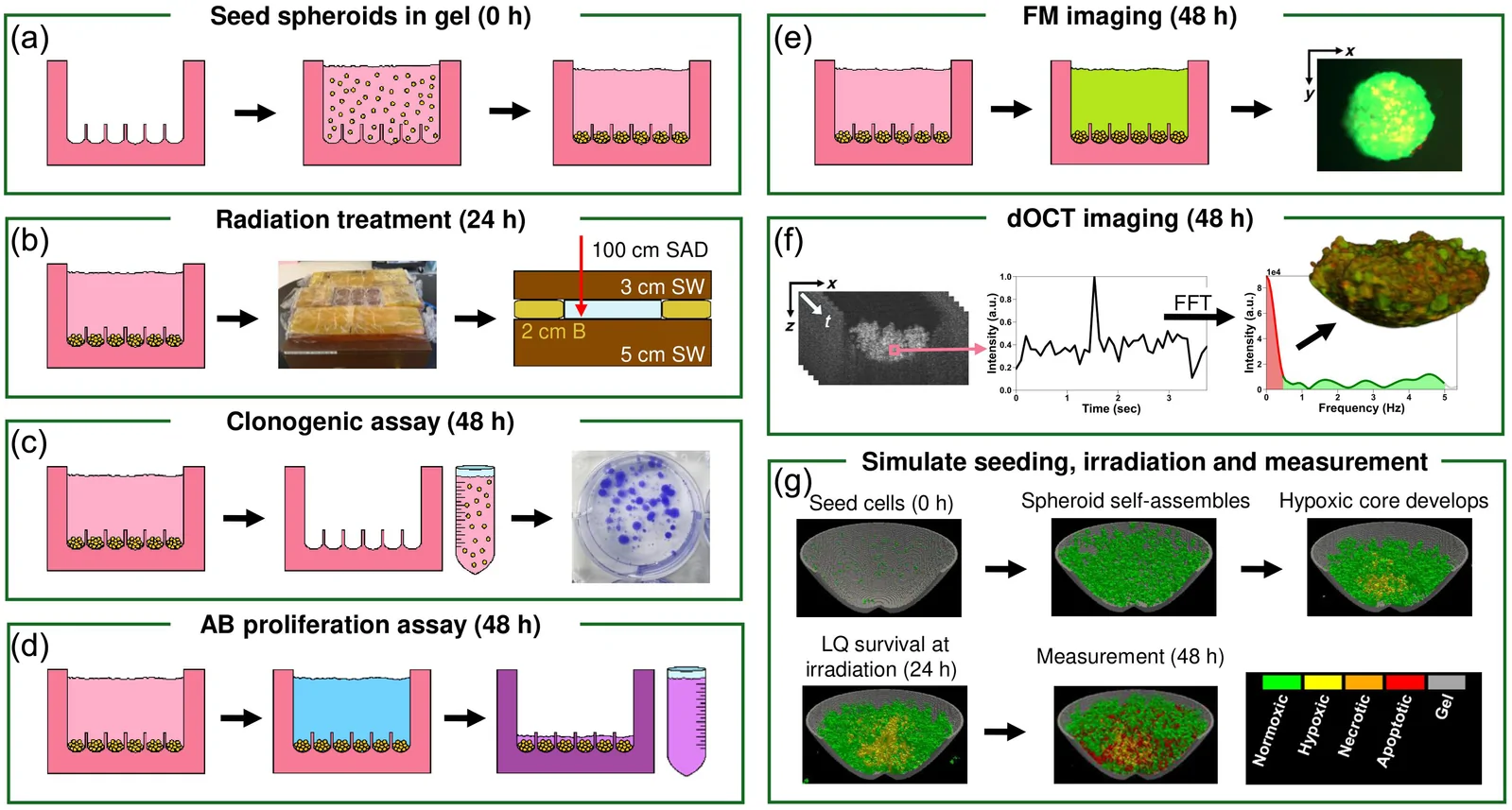
Schematic of experimental and simulated spheroid seeding, irradiation, and measurement. (a) Cells (yellow) seeded into agarose gels (pink) formed prostate tumor spheroids at the bottom of wells. (b) After 24 h, spheroids in 6-well plates were surrounded by Superflab bolus (yellow) and irradiated with 6-MV radiation at 5-cm depth in solid water (brown). Measurement was performed 24 h post-irradiation. (c) Clonogenic assay disaggregated spheroids for plating 2D cultures that were fixed and stained after 2 weeks. (d) In the AB proliferation assay, AB solution was added to the gels and then extracted for absorbance measurement. (e) For FM *en face* imaging, a solution of green calcein acetoxymethyl and red propidium iodide was added to the gels to label live and dead cells, respectively. (f) Volumetric dOCT imaging acquired 40 repeated frames per cross-section. Dynamic signals were extracted from intensity fluctuations via an FFT-based algorithm assigning red (<0.5  Hz) and green (0.5 to 5 Hz) frequency bands. (g) 3D agent-based simulation of spheroid seeding, irradiation, and measurement applied experimental LQ survival at treatment. SAD, source-to-axis distance; B, Superflab bolus; SW, solid water; AB, Alamar Blue; FM, fluorescence microscopy; dOCT, dynamic optical coherence tomography; FFT, fast Fourier transform; LQ, linear-quadratic model.

### 2.3 Biological Assays

#### 2.3.1 Clonogenic assay

Clonogenic assay was performed with monolayer and spheroid cultures 24 h post-irradiation to measure clonogenic survival [Fig. 1(c)]. To disaggregate spheroids for clonogenic plating, spheroids were aggressively flushed out of the wells with their surrounding media and transferred into a falcon tube for mechanical vortexing, followed by incubation for 2 h to allow cells to settle by gravity. The supernatant was then removed and replaced with at least twice the remaining volume in 0.25% trypsin-EDTA (Thermo Fisher Scientific). After incubation for 10 min, the trypsin solution was mechanically vortexed again and centrifuged for subsequent cell counting. For monolayer culture, cells were incubated in 0.25% trypsin-EDTA for 3 min then centrifuged for counting. Following cell counting, cells from each experimental condition were plated into 6-well plates at low densities and allowed to grow for ∼2 weeks. Colonies were then fixed with 4% formaldehyde in PBS for 1 h and subsequently stained with 0.5% crystal violet (Thermo Fisher Scientific) dissolved in water for 30 min. Stained colonies were counted, and the plating efficiency was calculated in live control samples as follows:

$$\mathrm{PE}=\frac{N_{\text{colonies}}}{N_{\text{plated}}},$$ (1)

where $N_{\text{colonies}}$ is the number of colonies counted, and $N_{\text{plated}}$ is the number of cells plated. Then, in treated samples, survival fraction (SF) was calculated as follows:

$$\mathrm{SF}=\frac{N_{\text{colonies}}}{N_{\text{plated}}\times \mathrm{PE}}.$$ (2)

The linear and quadratic parameters α and β were extracted by least-squares fitting of SF values for each treated dose d to the LQ model,

$$\mathrm{SF}=\exp(-\alpha d-\beta d^{2}).$$ (3)

#### 2.3.2 Alamar Blue proliferation assay

AB proliferation assay was performed 24 h post-irradiation to measure the average cellular proliferation within the tumor spheroids [Fig. 1(d)]. To achieve a final AB concentration of 5% within each 500-μL gel, 34.5  μL of AB (Sigma-Aldrich) and 156  μL of RPMI were added dropwise to each gel after careful removal of the culture medium in 10-μL increments. Samples were incubated for 3 h at 37°C with 5% ${\mathrm{CO}}_{2}$ in a humidified atmosphere. Following incubation, the absorbance intensity of the extracted solution was recorded at 570 nm using a BioTek Synergy H1 microplate reader. Three gels, each containing 81 spheroids, were measured for each experimental condition. Gels without cells were processed identically and measured to determine background absorbance. For each of the three measurements of spheroid-containing gels, the average background intensity was subtracted, and the resulting AB absorbance was normalized relative to the absorbance measured in the live control samples.

### 2.4 Fluorescence Microscopy

FM was performed 24 h post-irradiation to visualize cell death within spheroids using the live/dead cell viability assay (Sigma-Aldrich). Live cells were labeled using calcein acetoxymethyl (calcein-AM), which fluoresces green through intracellular processes.^51^ Dead cells were labeled using propidium iodide (PI), which fluoresces red upon binding with DNA or RNA that is inaccessible in live cells.^52^ After careful removal of culture medium, a solution containing 0.2875-μL calcein-AM, 1.15-μL PI, 95-μL RPMI, and 95-μL PBS was added to each gel. Samples were then incubated for 1 h at 37°C with 5% ${\mathrm{CO}}_{2}$ in a humidified atmosphere. Following incubation, gels were transferred to coverslip-bottomed Petri dishes and imaged using a Zeiss Axio Observer widefield microscope that was operated by the ZEN2 Blue Edition software and equipped with an Axiocam 506 mono camera. A Plan-Apochromat 63/1.4 Oil Ph3 M27 objective (Carl Zeiss Microscopy LLC, White Plains, New York, United States) was used to acquire fluorescent images, while an incubation plate (PECON, Erbach im Odenwald, Germany) maintained the temperature at 37°C during imaging.

### 2.5 Optical Coherence Tomography

Tumor spheroids were imaged with the LF-dOCT system 24 h post-irradiation [Fig. 1(f)]. A detailed description of the system design and performance can be found elsewhere.^49^ Briefly, the system employed a broadband superluminescent diode light source (cBLMD-T-850-HP, Superlum, Cork, Ireland) with a central wavelength of 842 nm and a full-width-at-half-maximum spectral bandwidth of 179 nm, achieving an axial resolution of 2.6  μm in air. A 5× microscope objective (M Plan APO, Mitutoyo, Kawasaki, Japan) provided a lateral resolution of 6.4  μm. The system operated at an image acquisition speed of 2000 frames per second and had ∼93-dB sensitivity, measured with an incident power of 3.5 mW at the image plane.

Volumetric dOCT images were acquired and processed following previously described methods.^53^ Each dOCT volume consisted of 400 B-scans, with each B-scan composed of 400 A-scans. The dOCT volume acquisition was divided into four sub-volumes, each imaged with 40 repetitions at a repetition rate of 10.7 Hz. OCT images of 15 tumor spheroids per experimental condition were acquired, with each spheroid imaged for ∼16  s during dOCT recording. Spheroids were maintained at 21°C without ${\mathrm{CO}}_{2}$ supply during imaging. All OCT and dOCT images presented for each experimental condition were generated from the same representative spheroid, whereas different spheroids were used for FM. For comparison with inverted FM, *en face* OCT and dOCT images were generated as maximum intensity projections over the bottom 240  μm of the spheroid.

Morphological OCT images were generated using a standard OCT image reconstruction process and are displayed in logarithmic scale with the surrounding gel masked. Volumetric OCT masks were analyzed numerically to estimate the volume $V_{\mathrm{sph}}$ and external surface area S of each spheroid, as well as the volume of internal gaps $V_{\text{gap}}$ enclosed by that surface area.

To generate dOCT images, a Hann window function was first applied along the time direction to the linearly scaled OCT intensity values at each pixel location. Following zero-padding of the data, a fast Fourier transform (FFT) was performed along the time axis.^54^^,^^55^ Next, integrals were calculated over two distinct frequency bands: a lower-frequency (“slow”) red band below 0.5 Hz and a higher-frequency (“fast”) green band between 0.5 and 5 Hz that were adapted from previous dOCT studies.^49^^,^^53^^–^^56^ After applying logarithmic scaling to each of the red and green dOCT channels, a small region of culture medium located above each spheroid in a central B-scan was selected. The sum of the mean and standard deviation of the intensity within this reference region was subtracted from each pixel location. To quantitatively analyze dynamic activity, a raw dynamic signal metric was computed for each masked spheroid as follows:

$$D_{\text{raw}}=\frac{\underset{\text{mask}}{\sum }I_{G}}{\underset{\text{mask}}{\sum }I_{R}+\underset{\text{mask}}{\sum }I_{G}}.$$ (4)

Here, $I_{R}$ and $I_{G}$ represent the intensity values of the red and green channels, respectively. The average raw dynamic signal measured in the formaldehyde-fixed spheroids was subtracted from the corresponding signal measured in live spheroids, and the resulting value was normalized relative to the live control as follows:

$$D_{\text{norm}}(d)=\frac{D_{\text{raw}}(d)-D_{\text{raw}}^{f}}{D_{\text{raw}}(0)-D_{\text{raw}}^{f}}.$$ (5)

Here, $D_{\text{raw}}(d)$ and $D_{\text{raw}}^{f}$ represent the raw dynamic signals measured in spheroids treated with radiation dose d (Gy) and formaldehyde-fixed spheroids, respectively. Due to attenuation with depth caused by spheroid density, the numerical analysis excluded any *en face* (lateral view) planes whose average intensity was lower than the median intensity across the entire volume. To display dOCT images, the masked red and green channels were independently normalized to those of a representative spheroid before a Gaussian FFT filter was applied with $\sigma_{R}=90$ and $\sigma_{G}=65$, respectively. Finally, a 3×3 median filter was applied to each channel before combining them into an RGB color image.

### 2.6 Simulation

For this study, we also developed an agent-based computer model of 3D tumor spheroid growth and radiation treatment using the software CompuCell3D^57^ [Fig. 1(g)]. Simulated time evolved according to a Monte Carlo method, which minimized the effective energy of the 3D lattice containing the spheroid. Each simulation began with a suspension of 1000 well-oxygenated normoxic tumor cells placed randomly inside the gel within a curved well described by

$$a[(x-\Delta x{)}^{2}+(y-\Delta y{)}^{2}]=z-\Delta z.$$ (6)

Here, as extracted from OCT images, $a=0.0025\;\mu m^{-1}$ and (Δx, Δy, and Δz) positioned the bottom of the well at the center of the bottom surface inside a simulation space measuring 810  μm across and 405  μm deep with 5-μm voxels. Initially suspended cells settle under gravity and self-assemble into a spheroid, driven by the energetic preference of live cells to be in contact with other live cells rather than the surrounding gel or culture medium. Oxygen, governed by a PDE, was maintained at a constant partial pressure in the culture medium and diffused through the spheroid with a diffusion coefficient approximately equivalent to half that of oxygen in water. Simulated cells consumed oxygen, and their growth rate depended on the local partial pressure of oxygen according to a Michaelis–Menten saturation curve. When a cell grew to a sufficient volume, it divided into two daughter cells of equal volume. Due to competition and limited oxygen diffusion within the spheroid bulk, normoxic cells reversibly became hypoxic when oxygen-deprived and irreversibly became necrotic upon severe oxygen deficiency. The simulation time scale was calibrated so that normoxic cells divided approximately every 24 h, and spheroid dimensions were comparable to those measured by longitudinal OCT imaging 24 h after seeding.^53^

Radiation treatment was simulated 24 h after seeding. At the treatment time, each cell within the spheroid probabilistically survived according to the LQ curve derived from spheroid clonogenic experiments. Cells killed by radiation irreversibly became apoptotic only upon attempted cell division. The simulation explicitly distinguished cell death due to necrosis by nutrient deprivation from radiation-induced apoptosis. However, necrotic and apoptotic cells behaved identically and, unlike normoxic and hypoxic cells, did not preferentially adhere to neighboring cells. Formaldehyde fixation was not explicitly simulated; instead, it was approximated by assuming all cells in the live control simulation became apoptotic at the time of treatment.

Numerical morphological and dynamic analyses of simulated live control spheroids and spheroids irradiated with 1, 2, 6, and 10 Gy were performed 24 h post-irradiation. Morphological metrics including spheroid volume, surface area, and internal gap volume were calculated voxel-wise using the 3D lattice. Simulated SF was calculated using Eq. (2) by estimating $N_{\text{colonies}}$ and $N_{\text{plated}}$ as the number of living cells and the total number of cells in the spheroid, respectively. Due to the stochastic nature of the Monte Carlo simulation, each condition was simulated 10 times to achieve reasonable coefficients of variation. Simulated SF and all simulated morphological metrics demonstrated coefficients of variation less than 10% and 1%, respectively. A detailed mathematical description of the simulation can be found in the supplemental document, which was adapted from Swanson.^58^

### 2.7 Statistics

All measurements are presented as averages with corresponding error bars or individual data points overlaid onto boxplots with medians demarcated by a thick line. Clonogenic SF error was estimated by Fieller’s theorem with a 95% confidence interval from a Poisson distribution, as described by Gupta et al.^59^ For comparison with alternative SF measurements, clonogenic SF was presented using the LQ fit. The upper (lower) LQ error bounds were calculated using LQ parameters, α and β, that were fit to experimental clonogenic SF values plus (minus) the SF error estimated by Fieller’s theorem. All other error bars represent standard deviation.

In the numerical morphological analysis, one-way analysis of variance (ANOVA) tests were performed to compare live control spheroids with spheroids irradiated with 10 Gy and those fixed with formaldehyde for each metric. If the omnibus ANOVA test was significant, independent Welch’s t-tests were conducted to compare each tested condition individually against the live control spheroids. In the numerical dynamic analysis, independent Welch’s t-tests were performed to compare the normalized SF measured by dOCT and AB proliferation assay for each dose of radiation. A significance level α of 0.05 was employed, and t-tests were Bonferroni-corrected. Statistically significant t-test results are indicated on the relevant plots with an asterisk.

## 3 Results

Figure 2 summarizes the volumetric OCT and simulated morphological analyses of live control, irradiated, and formaldehyde-fixed spheroids. The first column of Fig. 2 presents the spheroid volume, internal gap volume, and external surface area calculated voxel-wise in masked volumetric OCT images and simulation. As the radiation dose increased from 0 to 6 Gy, simulations predicted reductions in spheroid volume, internal gap volume, and surface area [Figs. 2(a)–2(c)]. All simulated morphological metrics then plateaued between 6 and 10 Gy, with 10 Gy significantly different from live control [Figs. 2(a)–2(c)]. Compared with the OCT measurements, simulated gap volumes and surface area values [Figs. 2(b) and 2(c)] were three and one orders of magnitude larger, respectively. Despite these differences in magnitude, the general trends of the morphological metrics observed by volumetric OCT were consistent with simulated results for radiation doses between 0 and 2 Gy [Figs. 2(a)–2(c)]. However, in the OCT analysis, the median spheroid volume, gap volume and surface area increased at 6 Gy, then decreased at 10 Gy, and further increased in formaldehyde-fixed spheroids [Figs. 2(a)–2(c)]. Notably, the surface area of formaldehyde-fixed spheroids was significantly larger than that of live control spheroids [Fig. 2(c)].

**Fig. 2:**
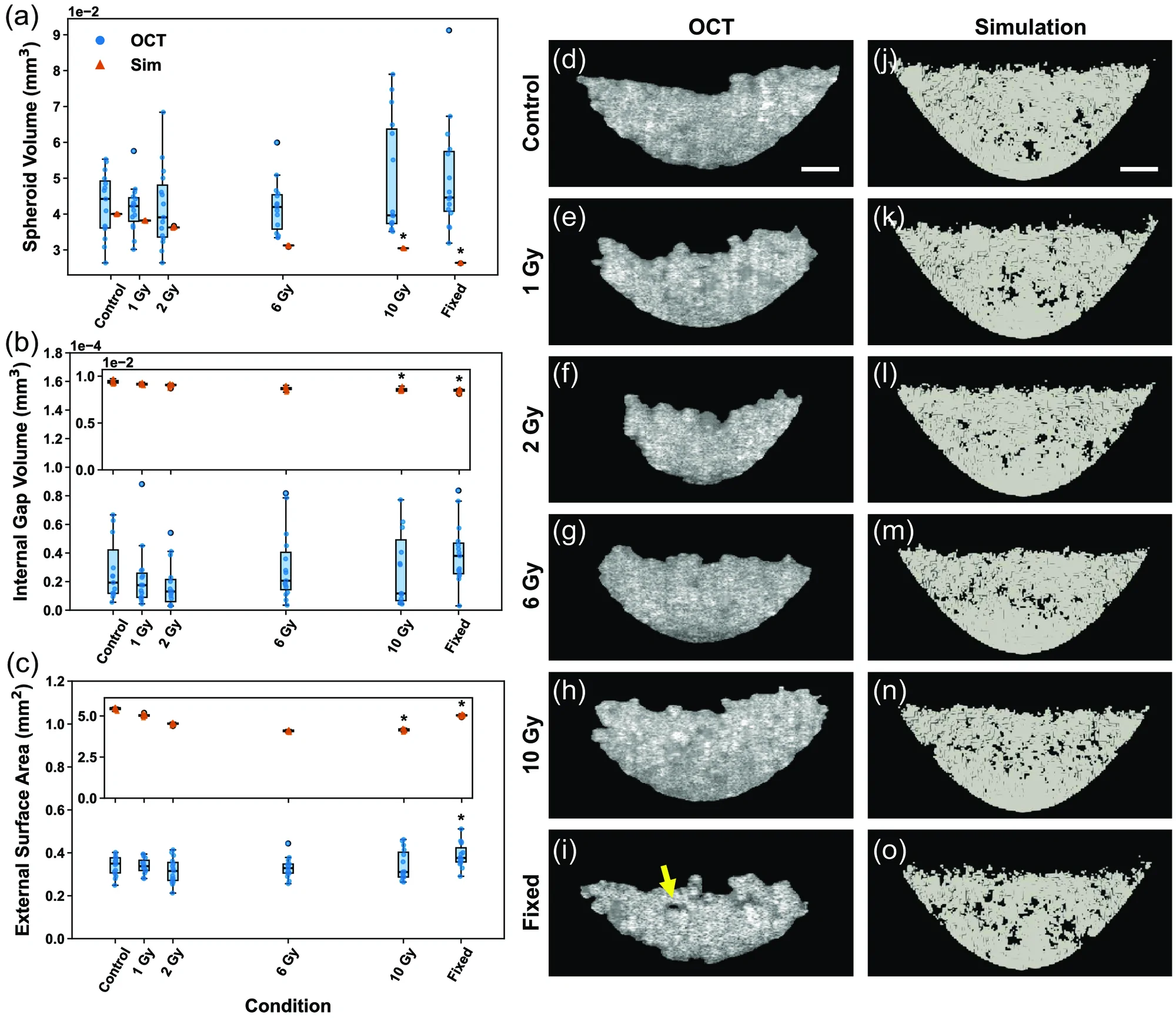
Morphological OCT and simulated analyses (first column) of live control, irradiated (1, 2, 6, and 10 Gy) and formaldehyde-fixed prostate tumor spheroids. Numerical analysis of (a) spheroid volume, (b) internal gap volume, and (c) external surface area. OCT-measured (blue circle) and simulated (orange triangle) values are presented with individual data points overlaid onto boxplots. Simulated data in panels (b) and (c) are inset. Asterisks indicate significant differences (10 Gy or fixed versus live control; Sec. 2.7). Central (second column) masked OCT B-scans and (third column) simulated visualizations of live control, irradiated (1, 2, 6, and 10 Gy), and formaldehyde-fixed spheroids. An internal gap is labeled with a yellow arrow. Sim, simulation. Scale bars: 100  μm.

The second and third columns of Fig. 2 show central cross-sectional masked OCT images and corresponding simulated visualizations, respectively. OCT images exemplify the smaller volume and surface area of the spheroid treated with 2 Gy [Fig. 2(f)] relative to those of the spheroid treated with 10 Gy [Fig. 2(h)], which is comparable in size to the live control [Fig. 2(d)]. An internal gap can be observed in the OCT image of a formaldehyde-fixed spheroid [Fig. 2(i), yellow arrow], which otherwise resembles the live control and irradiated spheroids. In contrast, simulated spheroids display largely uniform cross-sectional shapes and sizes across all radiation doses [Figs. 2(j)–2(n)]. However, simulated formaldehyde-fixed spheroids similarly contain more internal gaps than both control and irradiated spheroids [Fig. 2(o)].

Figure 3 summarizes the dynamic analysis. Clonogenic SF values for prostate cancer cells cultured in 3D spheroids and conventional 2D monolayers are presented in Fig. 3(a). Compared with 2D monolayer culture, cells cultured in spheroids demonstrated higher radioresistance with ∼6% and 2% clonogenic survival after irradiation with 6 and 10 Gy, respectively [Fig. 3(a)]. Figure 3(b) compares the SF of spheroids measured via normalized dynamic signals derived from masked volumetric dOCT images, normalized absorbance from AB proliferation assay, simulations, and clonogenic assay. Generally, SF values measured by dOCT were larger than those measured by AB, but the differences were not statistically significant; initially increasing slightly with radiation dose from live control, SF values measured by both methods then decreased and subsequently reached a maximum at 10 Gy [Fig. 3(b)]. In contrast, clonogenic and simulated SF values consistently decreased with increasing dose, with clonogenic SF decreasing at a faster rate [Fig. 3(b)]. Each spheroid was partitioned into “inner” and “outer” regions, and no appreciable differences in dOCT signal were observed (Fig. S1 in the Supplementary Material). The second and third rows of Fig. 3 show the volumetric dOCT images and simulated spheroids irradiated with 2, 6, and 10 Gy, respectively. In dOCT volumetric images, high-frequency (green channel) intensity was predominantly observed throughout the spheroid bulk across all radiation doses, reaching its maximum intensity in the spheroid treated with 10 Gy [Figs. 3(c)–3(f)]. Conversely, simulated volumetric images demonstrated a distinct hypoxic core in the live control spheroids [Fig. 3(g)], with an increasing proportion of apoptotic cells distributed throughout the spheroid bulk as radiation dose increased [Figs. 3(h)–3(j)].

**Fig. 3:**
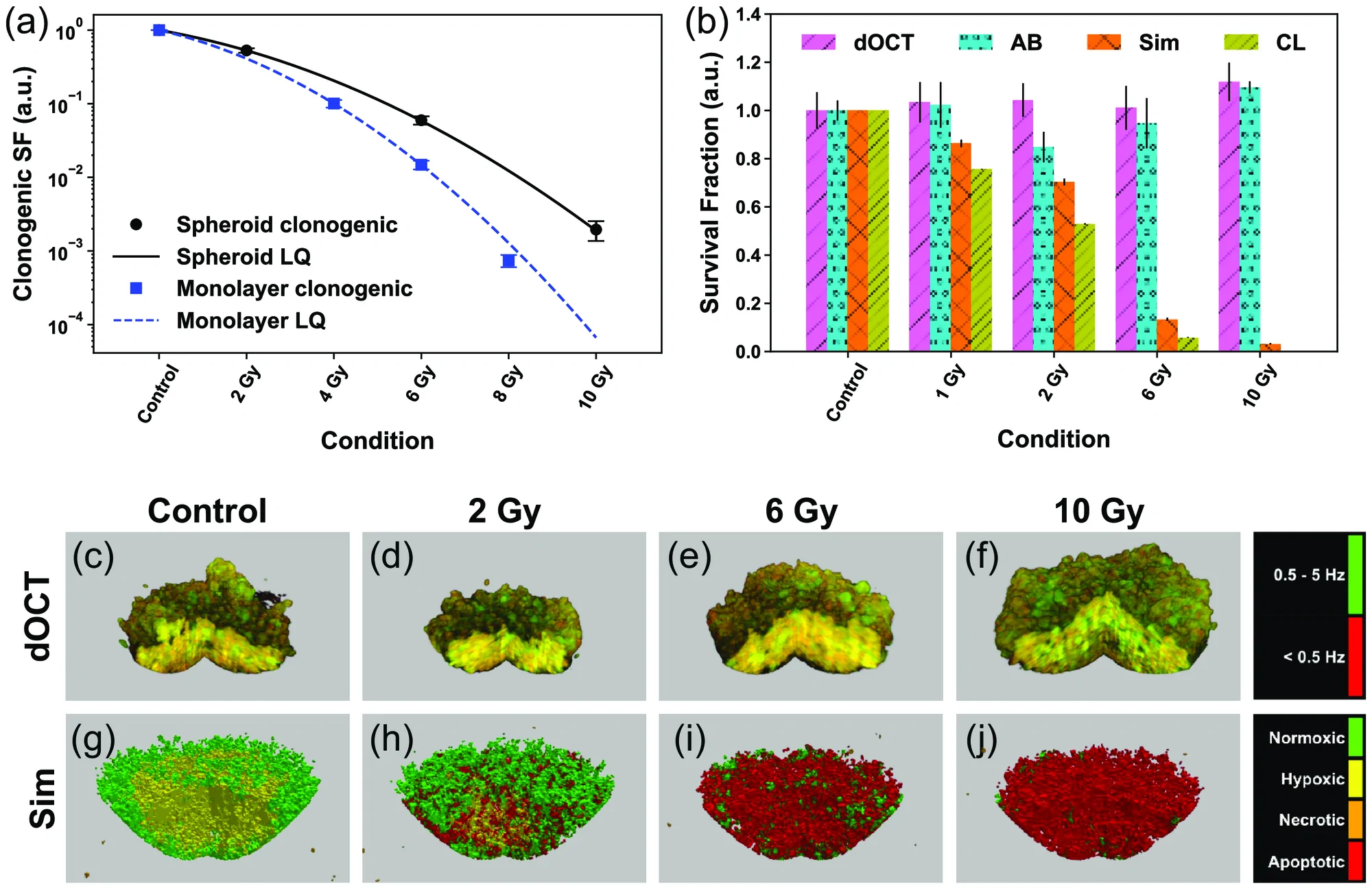
Dynamic analysis of live control and irradiated prostate tumor spheroids. (a) Clonogenic assay SF values with LQ model fit line for 3D spheroids (black circle, solid line) and 2D monolayer culture (blue square, dashed line). (b) SF of spheroids measured via normalized dynamic signal from volumetric dOCT images (purple / hatch), normalized absorbance from AB proliferation assay (blue circle hatch), simulated clonogenic assay (orange X hatch), and LQ fit from clonogenic assay (olive // hatch). Data shown are averages with error bars as described in Sec. 2.7. Volumetric dOCT images (c)–(f) and simulated visualizations (g)–(j) of live control and irradiated (2, 6, and 10 Gy) spheroids. A central slice has been cut out of the volumes. Colormaps for dOCT (red: <0.5  Hz; green: 0.5 to 5 Hz) and simulation (red: apoptotic; orange: necrotic; yellow: hypoxic; green: normoxic cells). SF, survival fraction; LQ, linear–quadratic; AB, Alamar Blue; Sim, simulation; CL, clonogenic.

Figure 4 compares OCT, dOCT, FM, and simulated *en face* images of live control, irradiated, and formaldehyde-fixed spheroids. OCT, dOCT, and FM enface images showed minimal variation between live control spheroids and those treated with radiation; however, live control and irradiated spheroids appeared significantly different from formaldehyde-fixed spheroids (Fig. 4). Individual cells could be resolved in OCT and dOCT enface images [Figs. 4(a)–4(l)]. In dOCT images, green channel intensity was higher at the spheroid periphery and increased slightly with increasing radiation dose in live control and irradiated spheroids [Figs. 4(g)–4(k)]. In contrast, formaldehyde-fixed spheroids exhibited ubiquitous red channel intensity with only a few individual cells appearing green [Fig. 4(l)]. In FM *en face* images, the live control and irradiated spheroids appeared predominantly green, with some central red fluorescence [Figs. 4(m)–4(q)]. However, after formaldehyde fixation, the FM image was dominated by red fluorescence and exhibited minimal central green fluorescence [Fig. 4(r)]. Unlike OCT, dOCT, and FM images, simulated spheroids were comprised of a progressively increasing proportion of red apoptotic cells as radiation dose increased [Figs. 4(s)–4(w)] and appeared entirely red following simulated fixation [Fig. 4(x)].

## 4 Discussion

### 4.1 Morphological Measurement of Radiation Effects

Simulations provide a platform for visualizing and precisely quantifying the outcomes of logical experiments conducted under perfectly understood, albeit oversimplified, biological conditions.^26^ Our 3D agent-based simulation assumed the spheroid grew within a symmetric paraboloid-shaped well given by Eq. (6) embedded in an agarose gel, with uniform oxygen diffusion and uniform oxygen consumption per unit volume of living cells. At the time of simulated radiation treatment, each cell survived irradiation with a probability equal to the SF given by the experimentally derived spheroid LQ curve shown in Fig. 3(a). Under these assumptions, we observed that simulated spheroid volume [Fig. 2(a)], internal gap volume [Fig. 2(b)], and external surface area [Fig. 2(c)] decreased with increasing radiation dose, subsequently plateauing between 6 and 10 Gy. According to the LQ model, the probability of cell death increased exponentially with increasing dose [Fig. 3(a)], resulting in a corresponding increase in the number of simulated apoptotic cells at 24 h after treatment [Figs. 4(s)–4(w)]. Simulated apoptotic cells failed to undergo cell division; thus, spheroids treated with lower doses exhibited larger volume and surface area due to a greater number of successful cell divisions occurring between treatment and analysis. The reduced internal gap volume in simulated spheroids treated with higher doses resulted from structural collapse caused by an increased proportion of non-adherent apoptotic cells. Despite a 10% decrease in simulated SF between 6 and 10 Gy [Fig. 3(b)], the corresponding morphological changes were negligible [Figs. 2(a)–2(c)] because the simulated spheroids irradiated with 6 Gy were already predominantly composed of apoptotic cells [Fig. 4(v)].

**Fig. 4:**
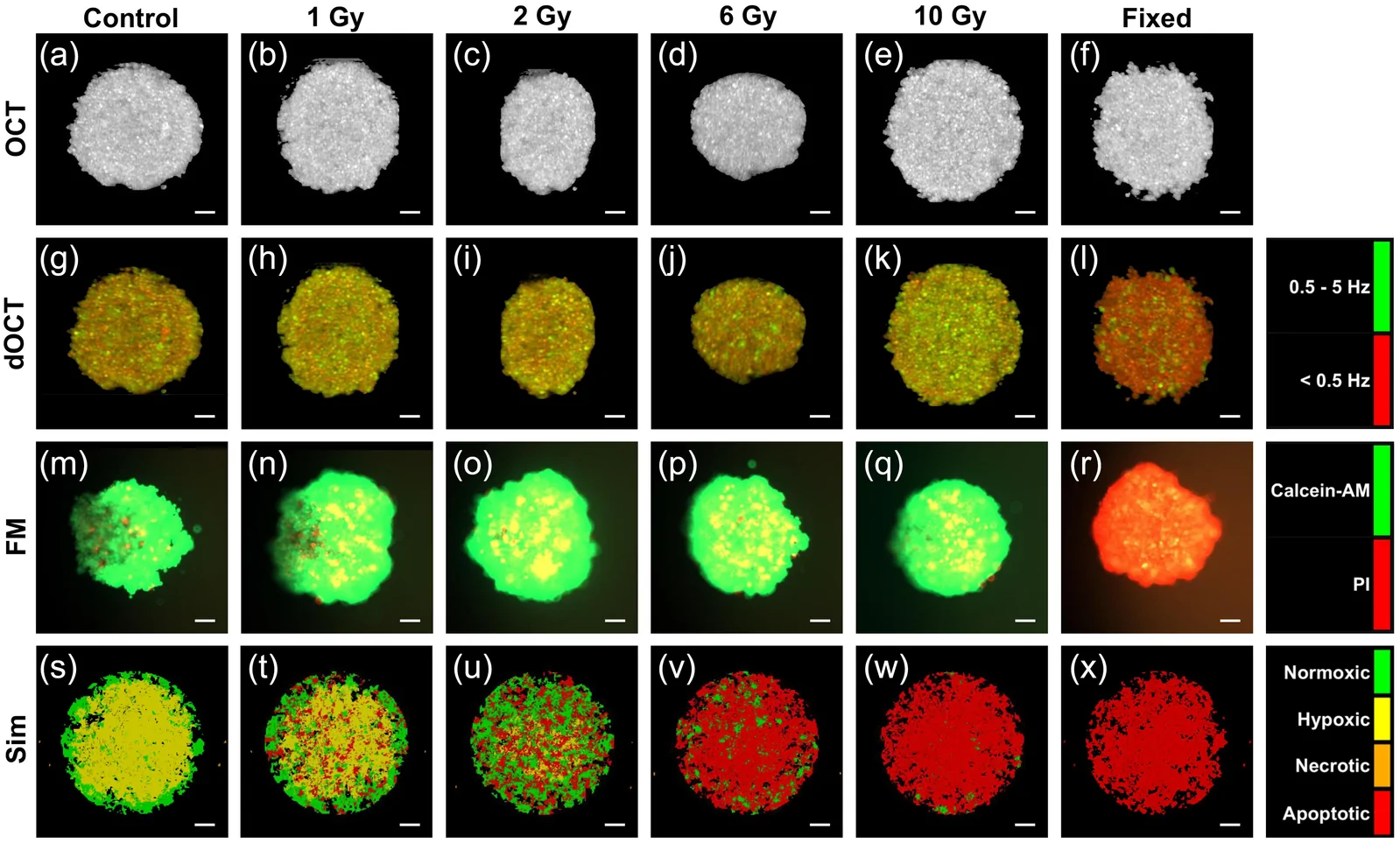
OCT maximum projection (a)–(f), dOCT maximum projection (g)–(l), FM (m)–(r), and simulated (s)–(x) *en face* images of live control, irradiated (1, 2, 6, and 10 Gy), and formaldehyde-fixed prostate tumor spheroids. Colormaps for dOCT (red: <0.5  Hz; green: 0.5 to 5 Hz), FM (red: PI; green: calcein-AM), and simulation (red: apoptotic; orange: necrotic; yellow: hypoxic; green: normoxic). FM, fluorescence microscopy; PI, propidium iodide; calcein-AM, calcein acetoxymethyl; Sim, simulation. Scale bars: 100  μm.

Simulated surface areas and internal gap volumes were significantly larger than those measured with volumetric OCT masks [Figs. 2(b) and 2(c)]. Simulations precisely quantified these metrics with a voxel resolution of 5  μm, whereas noise and an imperfect masking process complicated interpretation of the OCT measurements, despite our system demonstrating similar axial and lateral resolutions of 2.6 and 6.4  μm, respectively. The observed discrepancy in surface area illustrates the coastline paradox, wherein using higher resolutions to measure fractal-like surfaces, such as coastlines or spheroid surfaces, paradoxically increases the measured value. Rather than improving accuracy, the higher resolution of the simulation merely accounts for increasingly smaller surface features that the OCT mask smooths out. Furthermore, because OCT masks prioritize containing all cells within the spheroid, the smoothing process preferentially reduces internal gap volume and consequently overestimates spheroid volume. Underestimation of gap volume by OCT masking resulted in a greater discrepancy with the simulation than did overestimation of spheroid volume because the affected volume represents a substantially larger fraction of the total gap volume than of the total spheroid volume [Figs. 2(a) and 2(b)].

Despite these differences in absolute magnitude, morphological trends observed in simulated spheroids irradiated with low doses corresponded well with OCT analysis. Raitanen et al.^21^ similarly observed no significant size differences in PC3 spheroids irradiated with doses up to 8 Gy after 3 days. However, morphological discrepancies between simulation and OCT increased with increasing dose. Agreement was strongest for live control spheroids because the simulation was calibrated to spheroid dimensions measured in a longitudinal OCT study of untreated spheroids.^53^ With higher doses, the simplified mechanism of cell death and unrealistically instantaneous apoptotic process in the simulation diverged increasingly from the complex biological processes observed experimentally.

### 4.2 Clonogenic Measurement of Radiation Effects

In reality, radiation-induced cell death is neither instantaneous nor straightforward. Radiation damages DNA indirectly via reactive oxygen species and directly via ionizing tracks that create single-strand breaks and more lethal double-strand breaks.^8^^,^^60^^,^^61^ DNA damage triggers various signaling pathways that instigate DNA repair,^62^^,^^63^ the duration of which increases with the complexity of the damage and may extend beyond 24 h.^64^^,^^65^ Radiation exposure also induces G2/M phase arrest, delaying the onset of mitosis by prolonging the G2 phase of the cell cycle, thus preventing cell division with damaged DNA.^66^^,^^67^ Persistent cell cycle arrest leads to senescence, a state in which cells stop proliferating but can remain metabolically active.^68^ Cells with irreparable DNA damage may undergo mitotic catastrophe and subsequently die through some combination of apoptosis and necrosis.^8^^,^^61^^,^^62^^,^^68^ Thus, radiation results in clonogenic death by preventing cells from successfully performing cell division, although cells can be observed to remain metabolically active for some time after treatment.^21^^,^^69^

Consequently, the clonogenic assay remains the gold standard method for measuring cell survival after irradiation, as it directly assesses the clonogenic potential of individual cells. We measured higher clonogenic SF in prostate cancer cells cultured in 3D spheroids than in conventional 2D monolayer culture [Fig. 3(a)]. The improved clonogenic survival of spheroids compared with monolayer culture was first reported by Durand and Sutherland^19^ who attributed this phenomenon to increased cell-to-cell contact; it has since been documented across various cell lines.^20^^,^^21^^,^^69^^–^^73^ In addition, irradiated spheroids exhibit reduced DNA damage^21^^,^^72^ and apoptosis^73^ compared with monolayer culture. It has been suggested that the increased survival observed in spheroids may result from the presence of a quiescent hypoxic core, which has been widely documented.^20^^,^^22^^,^^23^^,^^69^^,^^74^ Clinically, hypoxia significantly contributes to tumor radio-resistance, in part due to the radio-sensitizing effect of oxygen.^75^^–^^77^ Cells have been observed to become quiescent under stress such as hypoxia,^23^^,^^78^ and quiescence itself is known to confer radio-resistance.^20^^,^^23^ However, PC3 cells form spheroids as loose aggregates that are not tightly adherent,^79^^,^^80^ and our FM results showed only a small necrotic core within live control spheroids [Fig. 4(m)]. Therefore, compared with other cell lines, the increased radio-resistance observed in PC3 spheroids relative to monolayer culture might be more plausibly explained by increased cell-to-cell contact rather than a quiescent hypoxic core.

### 4.3 Measurement of Death by Radiation Versus Fixation

Our study investigated multiple causes of cell death. At the time of radiation treatment, live control spheroids were also fixed with 4% formaldehyde to eliminate metabolic activity. Clonogenic assay demonstrated ∼2% clonogenic survival in spheroids irradiated with 10 Gy [Fig. 3(b)], and FM revealed widespread cell death in formaldehyde-fixed spheroids [Fig. 4(r)]. However, dOCT and FM images of control and irradiated spheroids both showed higher green channel intensity along the spheroid periphery with some central red channel intensity, whereas formaldehyde-fixed spheroids appeared almost entirely red in color [Figs. 4(g)–4(r)]. Both imaging techniques were applied 24 h after treatment, at a time when irradiated cells remained metabolically active and structurally intact.^21^^,^^62^^,^^69^ In contrast, fixation-induced cell death occurred quickly and efficiently. Thus, dOCT accurately detected the metabolically inactive, fixed spheroids as dynamically static, consistent with the extensive cell death observed by FM.

Morphologically, formaldehyde fixation preserved spheroid structure without significant tissue shrinkage by cross-linking proteins,^50^ effectively arresting spheroid morphology at the treatment time. Due to the simplified nature of our simulation, fixation was not directly simulated; instead, we approximated fixation by assuming all cells in live control spheroids at the treatment time were apoptotic [Fig. 4(x)]. At the time of treatment and fixation, 24 h after seeding, the spheroids were still condensing to become more tightly aggregated.^53^ This process is reflected in the larger OCT-measured spheroid volume [Fig. 2(a)], internal gap volume [Fig. 2(b)], and external surface area [Fig. 2(c)] of formaldehyde-fixed spheroids compared with live control, although the difference was only statistically significant for surface area. In contrast, the simulated formaldehyde-fixed spheroids had significantly smaller volume [Fig. 2(a)], internal gap volume [Fig. 2(b)], and external surface area [Fig. 2(c)] compared with live control spheroids. The morphological difference between OCT measurement and simulation is likely due to the simplified modeling of the complicated and prolonged process of spheroid aggregation. Consequently, simulated spheroids were smaller and more tightly aggregated at the time of treatment and fixation, and they remained so in the complete absence of simulated cell division and growth in the 24-h interval between fixation and analysis.

### 4.4 Comparison of Radiation Effect Measurements

We observed that the normalized SF values measured by dOCT were consistently higher than those measured by the AB proliferation assay at each radiation dose, although these differences did not reach statistical significance [Fig. 3(b)]. That said, the statistical power of the comparison between AB and dOCT measurements is limited by how AB requires each gel of 81 spheroids to be measured simultaneously as an aggregate measure. The dOCT method effectively distinguished intensity fluctuations due to cellular metabolism, clearly differentiating between control [Fig. 4(g)] and formaldehyde-fixed spheroids [Fig. 4(l)]. Similarly, AB reduction is a well-established indicator of cellular metabolism.^31^ However, the SF values measured by both dOCT and AB were substantially higher than those determined by clonogenic assay [Fig. 3(b)]. Although dOCT imaging and AB proliferation assay are more straightforward and efficient to perform than clonogenic assay, they capture cellular activity at only a single time point, which limits their detection of delayed radiation effects.^21^^,^^62^^,^^69^ Indeed, the numerous signaling pathways activated by radiation repair^62^^,^^63^ and subsequent cell death^68^^,^^81^^,^^82^ may increase cellular activity observed with dOCT and AB in the first 24 h following irradiation. High-frequency dOCT signals have been previously observed in cells experiencing stress.^83^ Furthermore, radiation has been reported to activate invasive and proliferative pathways mediated through epidermal growth factor receptor,^84^ integrins,^85^ and hypoxia-inducible factor,^86^ potentially increasing measured cellular activity. Moreover, dOCT measurements are sensitive not only to metabolic activity but also to physical changes in cell morphology and volume. Apoptotic cells undergo characteristic physical transformations, including shrinking, surface blebbing, and chromatin condensation (pyknosis), before nuclear fragmentation (karyorrhexis).^81^ In contrast, necrotic cells exhibit cell swelling, undergo nuclear disintegration (karyolysis), and ultimately degrade, releasing their contents.^68^^,^^81^ These physical transformations could explain the higher SF measured by dOCT compared with AB; however, the investigation of post-radiation physical changes such as blebbing and pyknosis would benefit from higher-resolution OCT imaging focused on specific regions rather than the entire spheroid volume. In addition, high-frequency motion detected by dOCT could also reflect motion artifacts unrelated to cellular behavior, such as Brownian motion, or external scanner and camera noise.

The simulated SF values were lower than those measured by dOCT and AB proliferation assay, despite all methods estimating instantaneous survival at a single time point 24 h post-irradiation [Fig. 3(b)]. The discrepancy is likely due to the simplified model of radiation-induced cell death. In our model, hypoxia indirectly delayed radiation effects by slowing cellular growth, delaying attempted cell division, and consequently delaying apoptosis. However, during simulated irradiation, hypoxic cells did not exhibit radio-resistance. In contrast, other models have explicitly included oxygen effects on radiation survival by modifying LQ survival with an oxygen saturation curve.^87^^,^^88^ Various investigators have also explicitly modeled the process of DNA damage repair,^88^^–^^90^ growth delay, and cell cycle arrest,^88^^,^^91^ as well as the effect of cell cycle phase on radiation survival.^92^^–^^96^ In this regard, agent-based models provide a particularly effective platform to characterize tumor heterogeneity and explore its implications in radiation treatment.

The clonogenic assay inherently integrates these complex biological factors into a binary probability of long-term clonogenic survival, which we simulated probabilistically at the time of treatment. However, simulated SF remained higher than clonogenic SF for all doses [Fig. 3(b)] because not all cells killed by radiation had attempted cell division and become apoptotic by the measurement time point, 24 h post-irradiation. In addition, our model assumed instantaneous cell death without explicitly modeling the duration of apoptosis, a process estimated to take ∼24  h.^82^ At the measurement time point, many cells classified as apoptotic in the simulation would realistically still be undergoing the physically and metabolically dynamic process of cell death, potentially contributing to the elevated SF values observed by both dOCT and AB proliferation assay.

### 4.5 Alternatives to the Clonogenic Assay

A rapid, simple, and reliable alternative to the clonogenic assay for measuring radiation survival has long been sought.^47^ The search for alternative methods is particularly salient for 3D spheroid culture, given the observation by Onozato et al.^20^ that the spheroid disaggregation required for clonogenic assay can itself influence radiation response. With some restrictions, proliferation assay measurements made over several days following irradiation have accurately estimated clonogenic survival in monolayer culture.^10^ For example, Buch et al.^30^ proposed a proliferation-assay-derived SF that demonstrated good correlation with clonogenic SF. Their method used the simple exponential growth of monolayer cell culture to quantify the radiation-induced growth delay.

Recent optical approaches provide complementary ways to monitor treatment response in intact 3D cultures. Fluorescence-based live-cell imaging offers molecular and spatial specificity for hypoxia and cell death after irradiation but generally requires exogenous probes or reporters.^33^ Bright-field analysis is simple and label-free but primarily reports organoid-scale morphology and sustained growth.^34^ Optical redox imaging provides a more metabolically specific readout through endogenous NAD(P)H and FAD autofluorescence and has detected heterogeneous treatment and chemoradiation responses.^35^^,^^97^ Quantitative oblique back-illumination microscopy provides label-free quantitative phase contrast at cellular resolution and has enabled continuous visualization of apoptosis and cell migration during therapy.^36^ Relative to these methods, the principal advantage of dOCT is its combination of label-free, depth-resolved volumetric imaging and activity-sensitive dynamic contrast. However, dOCT contrast is not molecularly specific and may include contributions from intracellular metabolism, structural motion, sample motion, and instrumental noise. dOCT is not universally superior; rather, it is best positioned as a complementary modality with a distinct balance of volumetric coverage and biological specificity.

Our study explored dOCT as another potential alternative alongside proliferation assay for assessing radiation effects in intact spheroids. One limitation of our work is that dOCT imaging was conducted at 21°C without ${\mathrm{CO}}_{2}$ supply, whereas samples were incubated with AB at 37°C with 5% ${\mathrm{CO}}_{2}$ in a humidified atmosphere for the proliferation assay. However, our previous study of untreated spheroid growth^53^ was performed with the same protocols and found excellent agreement between dOCT and AB measurements over a 2-week period. If our dOCT imaging environment influences the “fast” and “slow” frequency bands, we expect that the effect is consistent throughout our studies.

A larger limitation of our work is the single dOCT and AB measurement performed 24 h post-irradiation, which precludes the characterization of a radiation-induced growth delay. In our previous study^53^ of untreated spheroid growth, dOCT and AB signals did not exhibit exponential growth; instead, they initially increased before falling and repeating the cycle. We hypothesized that the cyclical signals arose from spheroids growing to a size large enough to limit nutrient access to the spheroid core, leading to widespread cell detachment that increased nutrient access and bolstered new growth. As such, we expect dOCT measurements in spheroids made over several days following irradiation to contain conflicting signals. In the short term, dOCT signals are expected to rise due to metabolism of surviving cells as well as radiative stress and the transient physical motion of necrosis or apoptosis. In the long term, dOCT signals are expected to fall once nutrients can no longer reach the spheroid core and once stressed irradiated cells undergo cell death. However, the radiation dose likely influences the inflection point at which dOCT signals begin to fall due to resource competition; higher doses are expected to lower initial metabolic signal and delay the inflection point while increasing initial radiative stress signal and producing a larger proportion of cell death with no dOCT signal in the following days.

It may be possible to extract the inflection point of the metabolic dOCT signal to quantify the radiation-induced growth delay under various conditions. However, we suggest an alternative to interpreting quantitative dOCT signals that are influenced by radiative stress and physical motion. Our single time point was selected to align with when spheroids were disaggregated and counted for the clonogenic assay 24 h post-irradiation. This short post-irradiation window minimizes cell division between irradiation and plating to ensure that each colony arises from a surviving irradiated cell. The clonogenic assay visually isolates cell division via sparse colony formation, but when surviving irradiated cells divide in intact spheroids, the progeny become visually indistinguishable from neighboring cells. We propose that tracking cell divisions via longitudinal volumetric dOCT as they occur within each intact spheroid may provide a better proxy for clonogenic survival. The strong dOCT signals we observed in irradiated cells in this study could provide improved contrast for visually identifying and tracking progeny to categorize each irradiated cell as colony-forming or not. This approach would (1) directly measure clonogenic survival in the spheroid environment, (2) avoid the unwanted influence of 2D cell geometry on radiation response, (3) leverage the volumetric spheroid data inaccessible to proliferation assays, (4) require an OCT imaging stage with an integrated incubator to maintain the temperature and ${\mathrm{CO}}_{2}$ for longitudinal imaging, and (5) benefit from higher-resolution sampling of a smaller area to improve cell division identification and visualize physical changes such as blebbing.

Numerous technological challenges must be addressed. Tracking individual cell divisions in intact spheroids would require sufficient intrinsic contrast and spatial resolution to separate adjacent cells, stable incubation-compatible longitudinal acquisition, robust three-dimensional registration as spheroids move and deform, and correction for depth-dependent attenuation and multiple scattering. In the present LF-dOCT system, deeper low-intensity planes were excluded from quantitative analysis, illustrating the penetration constraint, while the axial and lateral resolutions of 2.6 and 6.4  μm, respectively, may not consistently delineate cell boundaries throughout densely packed regions. Recent rolling-phase dynamic full-field OCT methods reduce phase noise and fringe artifacts and improve subcellular contrast,^98^ while swept-source-based dOCT imaging has also shown great potential for deep-tissue imaging.^99^

## 5 Conclusion

We employed a spectral-domain LF-dOCT system to acquire volumetric dOCT images of prostate tumor spheroids 24 h following radiation treatment. Morphological trends measured by volumetric OCT analysis aligned with simulations of a 3D agent-based model of radiation treatment whose parameters were informed by experimental clonogenic assay data. The effects of radiation measured using dOCT showed excellent quantitative and qualitative agreement with conventional biological techniques, AB proliferation assay and FM, respectively. Nonetheless, our work is limited by a single dOCT measurement performed 24 h post-irradiation. We identified a strong dOCT signal in irradiated spheroids, but our previous observation of cyclical spheroid growth complicates the interpretation of quantitative dOCT signals in multiple post-irradiation time point measurements. Instead, we suggest using longitudinal volumetric dOCT to track cell divisions in irradiated spheroids. This approach may offer a non-invasive alternative to the clonogenic assay, eliminating the need for spheroid disaggregation.

## Supplementary Material

10.1117/1.JBO.31.10.106004.s01

## Data Availability

The code used to process the OCT and dOCT images is publicly available on GitHub at https://github.com/skswanso/dOCT.git. The simulation code is also publicly available on GitHub at https://github.com/skswanso/CPM.git. The data underlying the results presented in this paper are not publicly available at this time but may be obtained from the authors upon reasonable request.
